# Natural language processing approach to model the secretion signal of type III effectors

**DOI:** 10.3389/fpls.2022.1024405

**Published:** 2022-10-31

**Authors:** Naama Wagner, Michael Alburquerque, Noa Ecker, Edo Dotan, Ben Zerah, Michelle Mendonca Pena, Neha Potnis, Tal Pupko

**Affiliations:** ^1^ The Shmunis School of Biomedicine and Cancer Research, George S. Wise Faculty of Life Sciences, Tel Aviv University, Tel Aviv, Israel; ^2^ Department of Entomology and Plant Pathology, Auburn University, Auburn, AL, United States

**Keywords:** type III secretion system, secretion signal, machine learning, natural language processing, effectors, pathogenomics

## Abstract

Type III effectors are proteins injected by Gram-negative bacteria into eukaryotic hosts. In many plant and animal pathogens, these effectors manipulate host cellular processes to the benefit of the bacteria. Type III effectors are secreted by a type III secretion system that must “classify” each bacterial protein into one of two categories, either the protein should be translocated or not. It was previously shown that type III effectors have a secretion signal within their N-terminus, however, despite numerous efforts, the exact biochemical identity of this secretion signal is generally unknown. Computational characterization of the secretion signal is important for the identification of novel effectors and for better understanding the molecular translocation mechanism. In this work we developed novel machine-learning algorithms for characterizing the secretion signal in both plant and animal pathogens. Specifically, we represented each protein as a vector in high-dimensional space using Facebook’s protein language model. Classification algorithms were next used to separate effectors from non-effector proteins. We subsequently curated a benchmark dataset of hundreds of effectors and thousands of non-effector proteins. We showed that on this curated dataset, our novel approach yielded substantially better classification accuracy compared to previously developed methodologies. We have also tested the hypothesis that plant and animal pathogen effectors are characterized by different secretion signals. Finally, we integrated the novel approach in Effectidor, a web-server for predicting type III effector proteins, leading to a more accurate classification of effectors from non-effectors.

## 1 Introduction

A large number of plant and animal bacterial pathogens use Type III Secretion Systems (T3SS), Type IV Secretion Systems (T4SS), and Type VI Secretion Systems (T6SS), to translocate bacterial proteins called effectors into host cells, thus promoting their pathogenicity (Green and Mecsas, 2016). While proteins encoding the secretion apparatus are relatively conserved among different bacterial clades, the effector repertoire is highly variable, even among closely related strains of the same species (Groisman and Ochman, 1996). Furthermore, experimental validation of putative effectors is both labor intensive and costly, motivating the development of bioinformatic algorithms for effector prediction. Detecting the complete repertoire of effectors encoded in a given pathogenic bacterial genome is a critical first step to elucidate the molecular mechanisms involved in the host-pathogen interactions.

Over a decade ago, others and we were the first to formulate the problem of effector identification as a machine-learning (ML) classification problem (Arnold et al., 2009; Burstein et al., 2009; Löwer and Schneider, 2009; Samudrala et al., 2009). Since then, numerous ML approaches have been applied to predict effectors for T3SS, T4SS and T6SS, for various animal and plant pathogenic bacteria (Yang et al., 2010; Niemann et al., 2011; Sato et al., 2011; Yang, 2012; Dong et al., 2013; Zou et al., 2013; Lifshitz et al., 2014; Burstein et al., 2015; Burstein et al., 2016; Hobbs et al., 2016; Teper et al., 2016; Ashari et al., 2018; Jiaweiwang et al., 2018; Nissan et al., 2018; Jiménez-Guerrero et al., 2020; Ruano-Gallego et al., 2021; Wagner et al., 2022).

Successful ML-based prediction relies on curated data to be used for training and validation, i.e., a set of known effectors as well as a set of non-effectors. Previous published work differed in the set of sequences used for training and testing, hampering fair comparisons among different ML classification tools. Moreover, numerous algorithms for ML-based classification were evaluated, including Naïve Bayes (Tay et al., 2010), Support Vector Machine (SVM) (Yang et al., 2010; Wang et al., 2013a; Goldberg et al., 2016), Random Forest (Yang et al., 2013), LightGBM (Wang et al., 2019), and recently deep learning approaches (Hobbs et al., 2016; Fu and Yang, 2019; Xue et al., 2019; Jing et al., 2021). Finally, some reports considered identifying effectors that share high sequence similarity with previously identified effectors as a success. In contrast, others specifically removed highly similar sequences from both train and test data, to emphasize the ability of their algorithm to detect novel effectors, i.e., effectors that share no sequence similarity with previously identified effectors.

Classic ML-tools extract different sets of features from each sequence. Common features used by many effector prediction algorithms include: (1) GC content; (2) Amino-acid composition of the N-terminus (see below); (3) Presence of sequence homology to other effectors, or to non-effectors. In essence, the ML classification task is to provide a function from each possible vector of features to a score, which reflects the likelihood that this sequence encodes an effector. Finding this function is computed based on training examples. These training examples are labeled, i.e., we know whether each sequence in this set encodes an effector or not. The function is then tested on labeled data that were not used for training, to evaluate the performance. Trained models can also be applied to unlabeled data for the task of discovering putative novel effectors, which are subsequently verified experimentally (Burstein et al., 2009; Burstein et al., 2015; Teper et al., 2016; Jiménez-Guerrero et al., 2020; Ruano-Gallego et al., 2021). However, for the purpose of comparing the accuracy of different classification algorithms, here we will only deal with labeled data.

Clearly, many features are highly informative for the task of differentiating type III effectors (T3Es) from non-effectors. For example, a common feature in ML-based T3E identification tools is sequence similarity to eukaryotic domains. As T3Es often interact directly with host proteins, they frequently have domains that resemble their host proteins, both in sequence and in structure. These domains are almost always found in eukaryotes only (Stebbins and Galaán, 2001; Desveaux et al., 2006; Jelenska et al., 2007; McCann and Guttman, 2008). While such features are highly informative for the identification of novel T3Es, it is clear that the interaction between the secretion apparatus and the T3E is not based on the presence of an eukaryotic domain. Extensive previous work has localized the secretion signal of T3Es to their N-terminal region. It is of high interest to elucidate the characteristics of this secretion signal, to better understand the biochemical mechanism by which the bacterial cell sorts its proteins to secreted versus non-secreted. A better understanding of the secretion signal will also improve ML-based methods that utilize N-terminus features as part of their prediction.

The secretion signal of T3Es was first shown to reside in their N-terminus by analyzing pathogenic *Yersinia* effectors. The N-terminus was shown to be both essential and sufficient for secretion. It was also shown that no clear sequence similarity exists between the N-termini of validated effectors, suggesting that the secretion signal is not a simple sequential motif (Michiels and Cornelis, 1991; Sory and Cornelis, 1994). Yet, the importance of specific amino acids for secretion was demonstrated both computationally and experimentally. For example, extensive mutagenesis of positions 2-9 of the *Yersinia* effector YopE has clearly shown the importance of serine residues within an amphipathic region for secretion (Lloyd et al., 2002). As stated above, efforts to characterize the secretion signal were often part of a more general task of developing ML algorithms for predicting novel effector proteins. Thus, for example, both the overall amino-acid composition within the N-terminus and the occurrence of specific residues at specific sites were considered as features in SIEVE, one of the first ML tools for predicting T3Es (Samudrala et al., 2009). Of note, in that work, different lengths of the N-terminal regions were considered, and it was concluded that accounting for more than 29-31 residues from the N-terminus does not contribute to the ability to correctly classify effectors from non-effectors. An optimum length of 30 residues was also concluded by Löwer and Schneider (2009). They implemented one-hot encoding and a sliding window approach to capture the amino-acid composition of the effectors N-termini. Another early ML work used a reduced alphabet, and suggested that positions 1-30 from the N-terminus were important for plant T3Es and 1-50 for animal T3Es (Arnold et al., 2009). In that work it was also suggested that secondary structure features had no significant contribution to prediction. A sliding window approach for characterizing the secretion signal was also suggested, accounting for such factors as hydrophobicity, polarity, and the occurrence of beta turns (Tay et al., 2010). Such an approach can potentially capture spatial variation of the signal along the sequence, yet it allows some flexibility with regard to the location. In contrast to Arnold et al. (2009); Yang et al. (2010) reported the benefit of including predicted secondary-structure information and solvent accessibility in addition to amino-acid composition for accurate prediction of T3Es. In that work it was further claimed that including k-mer based features did not contribute to prediction accuracy.

Following these initial efforts, additional representation of the amino-acid composition, combined with various ML algorithms and train and test data were developed (Sato et al., 2011; Wang et al., 2011; Wang et al., 2013a; Wang et al., 2013b; Dong et al., 2015; Goldberg et al., 2016; Hobbs et al., 2016; Cheng et al., 2018; Fu and Yang, 2019; Wang et al., 2019; Hui et al., 2020; Ding et al., 2021; Li et al., 2021; Yu et al., 2021). These studies differed in: (1) the way the sequences of effectors and non-effectors were encoded; (2) the selection of training and test data; (3) the algorithms used for classification; (4) the hyper-parameters tuning these classifiers. In addition, many of these works included features that are not related to the secretion signal, e.g., sequence similarity to host proteins and for plant pathogens and the existence of regulatory elements such as the PIP-box (Fenselau and Bonas, 1995).

In recent years, large scale pre-trained models such as BERT (Devlin et al., 2019) and GPT (Brown et al., 2020) have allowed for great progress in the field of NLP. Their success has led to the adoption of large scale pre-trained models in other domains, e.g., in the field of computer vision (Simonyan and Zisserman, 2014). Such pre-trained models enable the encoding of large amounts of domain knowledge into millions of learned parameters (Han et al., 2021). The extensive data analyzed during training should allow capturing many nuances of the domain, which otherwise would take experts years to discover. This approach of using automatic features to capture a problem space, greatly differs from classical ML approaches, which require careful extraction of meaningful features to adequately represent the data. Moreover, these hand-crafted features are often very task specific, making them useless in tasks that operate on a different problem space. In the context of our study, the pre-trained models were not developed in the context of T3Es. Yet, as we demonstrate, they are useful outside the immediate context for which they were developed. Finally, we note that the extensive model training in such cases is done only once.

Recently Facebook’s research team created such a large-scale pre-trained model on multiple sequence alignments (MSAs) (Rao et al., 2021). This “MSA-transformer” was pre-trained on a large database, 3.8TB in size, representing 28 million MSAs. Specifically, their data contained all the protein MSAs available in RefSeq (Li et al., 2021). The trained “MSA-transformer” can transform a user input MSA (or sequence) into a high-dimension vector, which ideally should capture the information in this MSA. In the original paper the utility of the transformer was demonstrated on two downstream tasks; protein contact prediction and secondary structure prediction. On both tasks the model accuracy was equivalent and sometimes even higher than state-of-the-art computational tools, showing the utility of this approach in biological domains as well.

In this work, we focused on ML-based analysis of the secretion signal of T3Es. We aimed to compare different methodologies to encode the secretion signal, on the same train and test data, using the same classifiers and hyperparameter tuning. As we aim to study the secretion signal harnessed within the N-terminal region of T3Es, we compared only methods that use the amino-acid sequence as input, i.e., we did not include features such as regulation and the presence of specific eukaryotic domains. In addition to previously-described features, we also aimed to test the utility of encoding the secretion signal using Natural Language Processing (NLP) approaches, specifically using large pre-trained models. Finally, we incorporated the optimal characterization of the secretion signal into Effectidor, our previously developed ML algorithm for predicting T3Es, which uses a host of features including regulation, spatial distribution of effectors within the genome, homology to known effectors and to non-effectors, to name but a few (Wagner et al., 2022).

## 2 Materials and methods

### 2.1 Data

Positive and negative datasets. For preparing the positive data, a total of 1,857 known effectors from plant and animal pathogens were first retrieved from the Effectidor web server (Wagner et al., 2022) at https://effectidor.tau.ac.il/T3Es_data/T3Es.faa. These data were filtered to remove closely related homologs by conducting a blastP search (all-against-all with an E value cutoff of 10^-4^) and randomly selecting a single representative from each connected component and by only considering effectors of length equal or higher than 100 amino acids. This resulted in a total of 641 positive effectors. From this dataset, we removed 84 *Xanthomonas* effectors to be served as the positive data for the “*Xanthomonas* dataset”. From the remaining effectors we randomly removed 60 effectors to serve as the positive set for the “test dataset”. The remaining 497 effectors serve as the positive “training data”. Thus, the entire data are comprised of three independent datasets: train data, test data, and *Xanthomonas*.

To prepare the negative data we first extracted all open reading frames (ORFs) of *Escherichia coli* K12 substr. MG1655. This strain does not encode for T3SS, and hence it is assumed that all the proteins encoded in this genome are not T3Es. To define a negative set for the “*Xanthomonas* dataset”, we searched for entries in *Xanthomonas campestris* strain 8004, using blastP with an E-value cutoff of 10^-4^, which show significant similarity to ORFs encoded in the *E. coli* strain. As stated above, as these proteins have a homolog in an *E. coli* strain that does not encode a T3SS, they are also assumed to be true negatives. The number of negative entries in the *Xanthomonas* dataset was 2,300. Note, the positive and the negative datasets are disjoint. To construct the negative data for the training and test datasets, we initially considered all *E. coli* proteins. To avoid any possible overlap between the negatives of the train and test data and the negative samples of the *Xanthomonas* data, we excluded from these data all queries that showed significant homology with any *Xanthomonas* ORF (using blastP with an E-value cutoff of 10^-4^). The remaining 2,490 entries were divided to 2,244 and 246 ORFs used as negatives for the training and test datasets, respectively. We note that the number of negative examples is an order of magnitude higher than the positive dataset. This situation better reflects the situation in empirical genomes, in which the number of effectors is a small fraction of the total number of proteins encoded within the genome.

All these datasets are available at: https://github.com/naamawagner/T3ES_secretion_signal_analysis/tree/main/data.

Positive datasets derived from plant and animal pathogens. The positive training and test sets were further divided to samples derived from plant pathogens and to samples derived from animal pathogens. This resulted in 268 and 40 samples derived from plant pathogens in the training set and test set, respectively, and 229 and 20 samples derived from animal pathogens in the training set and test set, respectively.

Dataset used for Effectidor runs. To evaluate the performance of Effectidor with and without the inclusion of the secretion signal score, we established data that included 80 *Ralstonia solanacearum* GMI1000 effectors (Peeters et al., 2013), and 2,661 non-effectors found by Effectidor based on similarity to *E. coli* K12 proteins.

### 2.2 Features considered

The following features were considered in this work. Of note, all these features were computed from the N-terminus of effectors and non-effectors. The length of the N-terminus considered is denoted as *m* below. The value of *m* was optimized as part of the cross-validation procedure on the training data (10 values of *m* were considered: 10, 20, 30, 40, 50, 60, 70, 80, 90, 100).

#### 2.2.1 Amino acid composition

Nine sets of features reflecting the amino acid composition of the peptide were considered:

##### 2.2.1.1 Euclidean distance to known effectors versus known non-effectors based on amino-acid composition (1 feature)

As a preprocessing step, the frequency of each of the 20 amino acids in the entire set of known effectors was computed, and similarly for the entire set of known non-effectors, in the training data. Let $aa_{e}^{i}$ and Let $aa_{ne}^{i}$ be the frequency of amino acid *i* in effectors and non-effectors, respectively $\left(\sum_{i=1}^{20}\;aa_{e}^{i}\;=\;\sum_{i=1}^{20}\;aa_{ne}^{i\;}=1\right)$ . Similarly, let $aa_{peptide}^{i}$ be the frequency of amino acid *i* in a given peptide that we aim to classify $\left(\sum_{i=1}^{20}aa_{peptide}^{i}=1\right)$ . We next computed the Euclidean distance between the amino acid composition vector of the peptide and the amino acid composition vector of the known effectors:


$$d_{e}(peptide)=\sqrt{\sum_{i=1}^{20}{\left(aa_{peptide}^{i}-aa_{e}^{i}\right)}^{2}}$$

Similarly, we computed the distance between the amino acid composition vector of the peptide and the amino acid composition vector of known non-effectors:


$$d_{ne}(peptide)=\sqrt{\sum_{i=1}^{20}{\left(aa_{ne}^{i}-aa_{peptide}^{i}\right)}^{2}}$$

The feature we considered is the difference between these two distances:


$$Feature_{1}=d_{e}(peptide)-d_{ne}(peptide)$$

##### 2.2.1.2 Euclidean distance to known effectors and Euclidean distance to known non-effectors (2 features)

In the description above, the considered feature is the difference between two distances. Instead of forcing the minus operation between these two distances, we can let the ML classifier decide how to optimally weight the two distances. Thus, in this approach, we consider two different features: *d_e_* (*peptide*) and *d_ne_* (*peptide*).

##### 2.2.1.3 Amino acid frequencies in peptide (20 features)

In the above descriptions, the individual amino-acid frequencies were forced into a distance formula. Here, we considered the 20 values of $aa_{peptide}^{i}$ as separate features, allowing the classifier to optimally weight them.

##### 2.2.1.4 Position-specific score matrix (1 feature). Hyperparameter: *C*

In the above descriptions, the locations of the amino acids along the peptide were ignored, i.e., shuffling the peptide sequence would not change the value of any of the above features. However, it is reasonable to expect that the location within the peptide is important. Let $n_{aa_{j}^{i}}(e)$ and $n_{aa_{j}^{i}}(ne)$ be the counts of amino acid *i* at position *j* in the set of known effectors and the set of known non-effectors, respectively. The probability of each amino acid at each position can be estimated by:


$$f_{aa_{j}^{i}(e)}=\frac{n_{aa_{j}^{i}(e)}}{\sum_{k=1}^{20}n_{aa_{j}^{k}(e)}}$$

And similarly, for $f_{aa_{j}^{i}(ne)}$ . This computation provides a probability for each amino acid in each position, a data structure called position-specific score matrix (PSSM) or position weight matrix (PWM). It is common to add pseudo-counts to avoid zero probabilities (Durbin et al., 1998). We thus modify the computation of the PSSM to include a pseudo-count.


$$f_{aa_{j}^{i}(e)}=\frac{n_{aa_{j}^{i}(e)}+C}{\sum_{K=1}^{20}(n_{aa_{j}^{K}(e)}+C)}$$

In our implementation, we used *C* = 1.

Next, to score a peptide, let *p_1_, p_2_…, p_m_* be the amino acids in the peptide in each of its *m* positions. The contribution of *p_1_* to the score is its probability to appear in the first position, based on the PSSM. Formally, the entire score of the peptide when compared to the PSSM of effectors is:


$$PSSM_{Score}(e)=\sum_{k=1}^{m}\log(f_{aa_{k}^{Pk}(e)})$$

And similarity, for non-effectors:


$$PSSM_{Score}(ne)=\sum_{k=1}^{m}\log(f_{aa_{k}^{Pk}(ne)})$$

The feature we consider is the difference between the two PSSM scores:


$$PSSM_{Score}=PSSM_{Score}(e)-PSSM_{Score}(ne)$$

##### 2.2.1.5 Position-specific score matrices (2 feature). Hyperparameter: *C*

As in the above, it may be more informative to consider the two PSSM scores, *PSSM_Score_* (*e*) and *PSSM_Score_* (*ne*) as two separate features.

##### 2.2.1.6 Position-specific score matrices per position (*m* features). Hyperparameter: *C*

It may be more informative to consider the score of each position as a separate feature. Thus, in this representation, we consider the following *m* features: $\log(f_{aa_{j}^{Pj}(e)})-\log(f_{aa_{j}^{Pj}(ne)})$ , where *j* = 1,…,*m*.

##### 2.2.1.7 Position-specific score matrices per position (2*m* features). Hyperparameter: *C*

In the above descriptions, the individual PSSM scores per each position were combined into a single feature by a subtraction operation. Here, we consider each score as an individual feature, thus allowing the classifier to optimally weight each of them. The 2*m* features in this case are the *m* values of $\log(f_{aa_{j}^{Pj}(e)})$ and the *m* values of $\log(f_{aa_{j}^{pj}(ne)})$ , where *j* = 1,…,*m*.

##### 2.2.1.8 One-hot encoding per position (20*m* features)

In One-hot encoding each amino acid in each position is represented as a binary vector of size 20, where each entry is 0 if the amino acid is absent and 1 if present. These vectors are than concatenated to create a 20*m* representation of the entire sequence, where *m* is the length of the peptide. Each coordinate of this vector is considered as a separate feature.

##### 2.2.1.9 One-hot encoding with a sliding window (20w features); Hyperparameter: *w, l*

The above algorithm can be trivially extended to overlapping windows of size *w*. When using such a sliding window approach, the entire peptide sequences is divided to overlapping windows. The degree of overlapping is defined by the offset parameter *l*, the number of characters in the left window that are not included in the right window. In this work, we used *l* = 1. In such an approach each peptide contributes several windows to the learning (and for the testing) and each such a window is encoded by a vector of size 20w features. In other words, the trained classifier predicts for each sequence of length w whether or not it is a T3E. Once a new sequence is provided, the trained classifier predicts for each of its *m*-*w*+1 windows whether or not it is part of a T3E. If the majority of windows are predicted to be T3E, then the entire sequence is predicated to be T3E (Löwer and Schneider, 2009).

#### 2.2.2 Hidden Markov Model (1 feature)

Hidden Markov models (HMMs) are probabilistic models. Each hidden state generates columns based on probabilities similar to a PSSM matrix, i.e., it emits characters (in our case, amino acids) based on a specific frequency distribution. The entire sequence is modeled by a Markov process over the hidden states, i.e., the sequence is represented by an ensemble of hidden states. Here, we trained the HMM model using the Baum-Welch algorithm (Rabiner, 1989), which is an expectation-maximization (EM) algorithm that iteratively improves the data likelihood function until convergence. The number of hidden states was determined using a 3-fold cross-validation. To this end, the entire set of training data was randomly split to three folds. The HMM model was trained on the positive sequences of two of the folds with each possible value of number of hidden states between 1 and 20. The obtained HMM model for each number of hidden states was then used to evaluate the log-likelihood of each sequence in the third fold (both positive and negative). The performance of a classifier with a single feature (the HMM’s log-likelihood score) was used to evaluate the performance (using the MCC value) of each possible number of hidden states. This was repeated three times, each time a different fold was used for evaluation. The number of hidden states that was selected was the one that yielded the highest average MCC. After the number of hidden states was determined (the optimum was 10), the HMM was trained on the entire set of positive training data. The score of this HMM for each sequence in the test data was treated as a feature for classification.

#### 2.2.3 LSTM model

Long short-term memory (LSTM) (Hochreiter and Schmidhuber, 1997) networks are a form of recurrent neural networks designed to process sequential data. The data must be segmented into tokens, and in the case of protein sequences usually each amino acid is referred to as a token. These networks operate on raw sequence data and learn how to represent these data as part of their training process. LSTM networks are often used for translation between two languages. To adapt this translation task for classifying proteins to either T3E or non-effector, the model was trained to translate the protein sequence into a language that has only two words, yes and no, corresponding to whether the sequence is a T3E or not, respectively. Here we used an LSTM with an encoder-decoder architecture, each having a single RNN layer with a hidden state of size 512 from the “fairseq” Python package (Ott et al., 2019).

#### 2.2.4 Using Facebook’s “MSA transformer” for classification – LME (1,280 features)

Facebook’s “MSA transformer” allows to leverage a very extensive training process to extract meaningful features from protein sequence data (Rao et al., 2021). Facebook’s “MSA transformer” receives as input an alignment in FASTA format and here we used as input an “alignment” of a single sequence. The output is a set of 1,280 weights extracted from the last (34^th^) layer of the encoder. These weights are used by the neural network to encode the sequence, and can be used as a 1,280-dimensional feature vector.

#### 2.2.5 Features from Hobbs et al. (17 features)

Hobbs et al. (2016) suggested a set of features that can potentially classify effectors from non-effectors. We tested whether the inclusion of these features improves classification accuracy. Specifically, the following sets of amino acids are defined: (1) Tiny: A, C, G, S, T; (2) Small: A, C, D, G, N, P, S, T, V, B; (3) Aliphatic: A, I, L, V; (4) Aromatic: F, H, W, Y; (5) Polar: D, E, H, K, N, Q, R, Z; (6) Non-polar: A, C, F, G, I, L, M, P, V, W, Y; (7) Charged: D, E, H, K, R, B, Z; (8) Basic: H, K, R; (9) Acidic: D, E, B, Z. Each such a set defines a single feature, which is the fraction of positions in which one of the amino acids in the set is present. These features were implemented in Python.

The following features were implemented using the Biopython package “ProteinAnalysis”: (10) “Charge”, which measured the total charge of the peptide (in pH = 7); (11) A_280_ molar extinction coefficient, which predicts the light absorbance of the protein in 280 nm; (12) Isoelectric point; (13) Instability index; (14) Aliphatic index; (15) GRAVY Score; (16) Molecular weight. (17) “Probability of expression in inclusion bodies (PEPIB)”, which was implemented in Python based on Ahuja et al. (2006).

### 2.3 ML model

Protein sequences were classified to either effectors or non-effectors using LightGBM, a decision-tree classifier with gradient boosting (Ke et al., 2017), as implemented in the Python package lightgbm. The following LightGBM hyperparameters were optimized using ten-fold cross validation on the train data: type of boosting algorithm (‘gbdt’, ‘dart’, ‘rf’, ‘goss’), number of leaves in each tree (10, 30, 50, 100, 200), tree depth (10, 100, 1,000, infinite), learning rate (0.1, 0.05, 0.005), number of tree estimators (10, 50, 100, 200, 1,000), alpha (0, 0.5, 1, 3, 10, 100), lambda (0, 0.5, 1.5, 3, 100, 500, 1,000, 1,200), and is-balanced (True/False). The is-balanced hyperparameter controls weights assigned to each class (in our case, effectors versus non-effectors), and may be highly important for unbalanced datasets.

### 2.4 Performance evaluation methods

Several scoring methods were used to evaluate performance. As the data are unbalanced, i.e., the number of negative samples is an order of magnitude higher than the number of positive samples, traditional scoring methods such as accuracy or the Area Under the Curve (AUC) are not well suited. Instead, the Area Under the Precision-Recall Curve (AUPRC) and Matthew’s Correlation Coefficient (MCC) are more suitable for unbalanced data. While the AUPRC score is used for probabilistic predictions, the MCC score is used for binary predictions, and is calculated with the following formula:


$$MCC=\frac{TN\times TP-FN\times FP}{\sqrt{(TP+FP)(TP+FN)(TN+FP)(TN+FN})}$$

Where TN (True Negative) is the number of non-effectors correctly classified as non-effectors, TP (True Positive) is the number of effectors correctly classified as effectors, FN (False Negative) is the number of effectors misclassified as non-effectors, and FP (False Positive) is the number of non-effectors misclassified as effectors. In cases where the AUPRC was impossible to compute, i.e., in deterministic prediction rather than a probabilistic one, the F1 score was used. The F1 score is calculated using the following formula:


$$F1=\frac{2\times precision\times recall}{precision+recall}$$

While precision and recall are given in the following formulas:


$$precision=\frac{TP}{TP+FP},\;recall=\frac{TP}{TP+FN}$$

### 2.5 Integrating the signal score into the Effectidor web server

Our results below show that the LME model (1,280 features) together with the 17 features from Hobbs et al. (2016), provides the best classifier based on the secretion signal. We term the lightGBM classifier, which was trained on the entire data based on these features, “trained LME”. The hyperparameters of this model were the same as those found to yield the best performance using cross validation on the training data. This trained LME model provides for each possible protein a score that reflects its propensity to harbor a type III secretion signal within its 100 N-terminal positions. This score was added as an optional feature within the Effectidor web server (Wagner et al., 2022).

**Figure 1:**
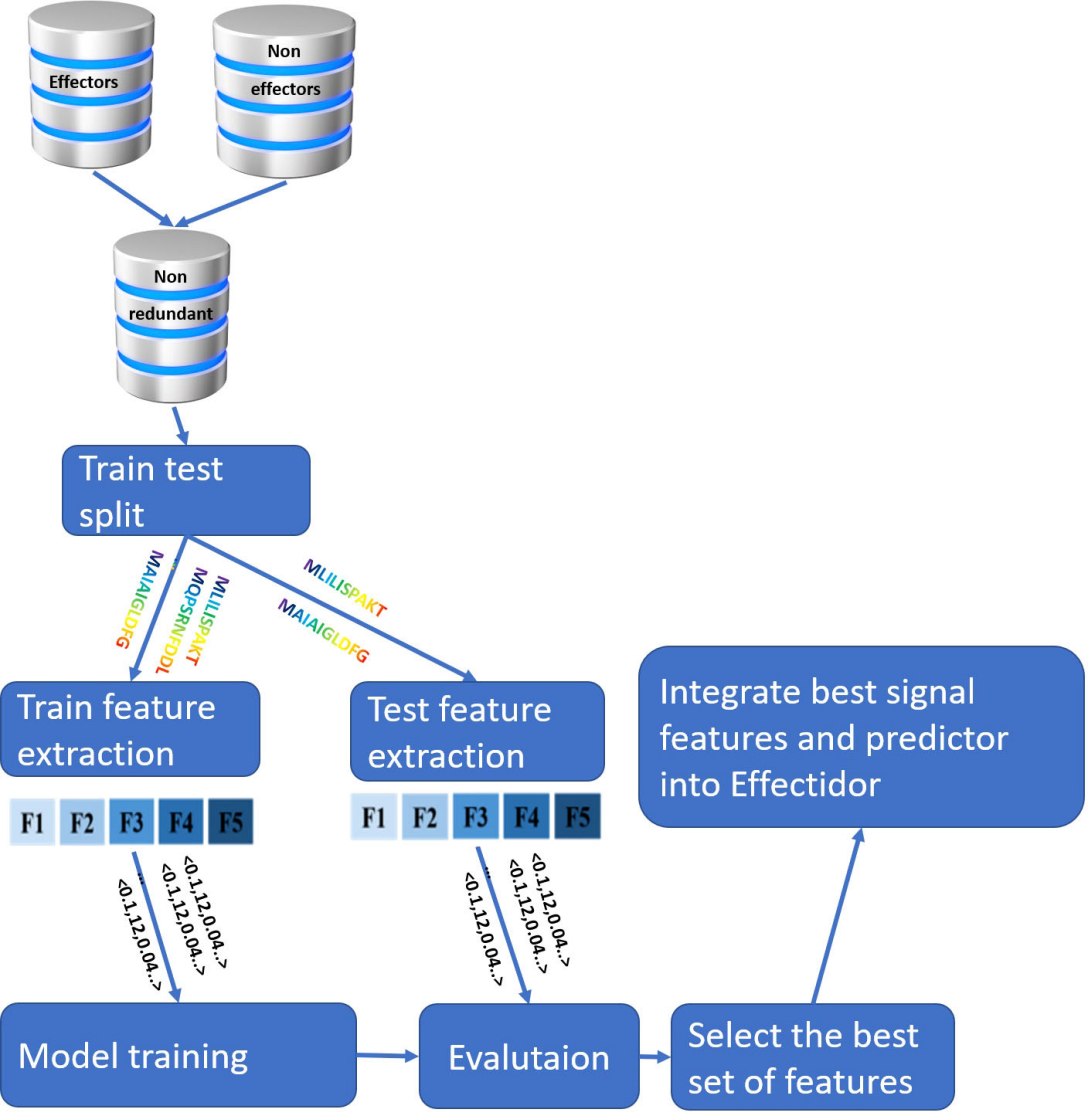
Overview of the pipeline implemented in this work. The input for the feature extraction are the N-terminal protein sequences, {F1,..., F5} represent different features, and the input to the model training and evaluation are the vectorial representations of the protein samples, as represented by the features.

An overview of the entire pipeline implemented in this work is available in **Figure 1**.

## 3 Results

### 3.1 Performance of all algorithms

**Figure 2:**
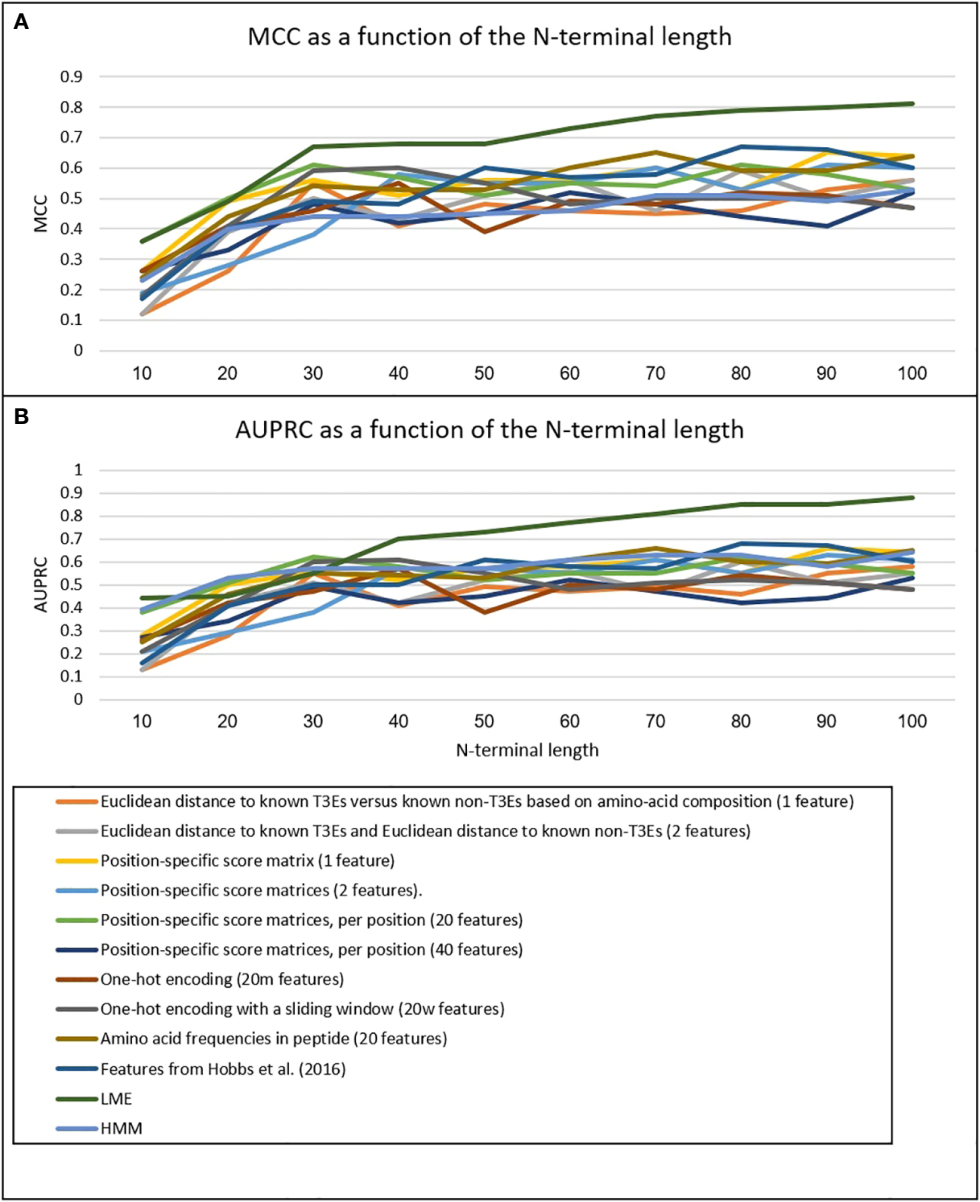
MCC **(A)** and AUPRC **(B)** of the different methods as a function of the N-terminal length. Scores are the mean scores of 10-fold Cross-Validation on the training data.

We have implemented three new ML-based approaches to model the secretion signal of T3Es: LME, LSTM, and HMM (see Methods). Our results clearly show that on both testing data, the accuracy is highest for LME, and lowest for HMM (**Table 1**). The LME accuracy was highest when all 100 amino acid residues of the N-terminus were considered (**Figure 2**). In all three methods, the accuracy was higher for the test dataset compared to the *Xanthomonas* dataset (for the LME methodology, the MCC and AUPRC were 0.81 and 0.88 for the test dataset, respectively, and 0.71 and 0.77 for the *Xanthomonas* datasets, respectively).

**Table 1:** The performance of the three methods proposed in this work. MCC and AUPRC (in parenthesis) on the test datasets (“test dataset” and “*Xanthomonas* dataset”).

	Hidden Markov Model	LSTM	LME
**Test dataset**	0.43 (0.4)	0.63 (0.7)	**0.81 (0.88)**
***Xanthomonas* dataset**	0.12 (0.05)	0.37 (0.44)	**0.71 (0.77)**

On the LSTM methods, instead of AUPRC, the F1 score is given, as it was a deterministic prediction. These results were obtained by analyzing the 100 N-terminal amino acids. In bold are the best scores for each of the test datasets.

We then compared these methods to nine alternative methods to model the secretion signal (**Table 2**). The best of these methods performed substantially poorer compared to the LME method (**Figure 2**). For the test dataset, the highest accuracy among these nine methods was obtained using the 20 amino acid frequencies as features, yielding an MCC and AUPRC values of 0.65 and 0.71, respectively. For the *Xanthomonas* dataset, the best performing method was position-specific score matrices per position (*m* features), with an associated MCC and AUPRC values of 0.45 and 0.5, respectively. These results clearly show that our proposed novel LME methodology is well suited for modeling the secretion signals of T3Es.

**Table 2:** The performance of alternative methods to model the secretion signal.

	Test dataset	*Xanthomonas* dataset
Euclidean distance to known T3Es versus known non-T3Es based on amino-acid composition (1 feature)	0.56 (0.6)	0.41 (0.5)
Euclidean distance to known T3Es and Euclidean distance to known non-T3Es (2 features)	0.54 (0.58)	0.32 (0.28)
Position-specific score matrix (1 feature)	0.65 (0.7)	0.34 (0.45)
Position-specific score matrices (2 features).	0.61 (0.66)	0.31 (0.4)
Position-specific score matrices, per position (20 features)	0.53 (0.59)	**0.45 (0.5)**
Position-specific score matrices, per position (40 features)	0.5 (0.52)	0.31 (0.42)
One-hot encoding (20*m* features)	0.5 (0.51)	0.24 (0.2)
One-hot encoding with a sliding window (20*w* features)	0.5 (0.49)	0.24 (0.3)
Amino acid frequencies in peptide (20 features)	**0.65 (0.71)**	0.41 (0.5)
Features from Hobbs et al. (2016)	0.61 (0.69)	0.44 (0.49)

MCC and AUPRC (in parenthesis) on the test datasets. These results were obtained by analyzing the 100 N-terminal amino acids. In bold are the best scores for each of the test datasets.

### 3.2 Testing feature combinations

We next tested the hypothesis that the LME method can be further improved by integrating several features. As the various models for amino-acid composition (see section 2.2.1) are very similar, for the combination analysis we selected the best performing method among them, i.e., amino-acid frequencies. We tested seven combinations of feature groups. Results on the test dataset as well as the *Xanthomonas* dataset show that combining different groups of features had a marginal impact on accuracy: on the test dataset, the best performing combination increased the MCC and AUPRC scores from 0.81 and 0.88 to 0.83 and 0.91, respectively. On the *Xanthomonas* dataset, the MCC and AUPRC scores were improved from 0.71 and 0.77 to 0.72 and 0.87, respectively (**Table 3**). We conclude that the major improvement in modeling the secretion signal stems from the proposed LME method.

**Table 3:** The performance of combinations of top-scoring features from each group of features.

Feature combination	Test dataset	*Xanthomonas* data
Amino acids frequencies + LME	0.81 (0.83)	0.70 (0.72)
LME + HMM	0.8 (0.8)	0.68 (0.7)
LME + Features from Hobbs et al. (2016)	**0.83 (0.91)**	**0.72 (0.87)**
Amino acids frequencies + LME + HMM	0.81 (0.81)	0.7 (0.7)
Amino acids frequencies + LME + Features from Hobbs et al. (2016)	0.8 (0.81)	0.7 (0.72)
HMM + LME + Features from Hobbs et al. (2016)	0.82 (0.85)	0.71 (0.73)
Amino acids frequencies + LME + HMM + Features from Hobbs et al. (2016)	0.82 (0.83)	0.72 (0.72)

The results were obtained on the test datasets by analyzing the 100 N-terminal amino acids. The scores are MCC and AUPRC (in parenthesis). In bold are the best scores for each of the test datasets.

### 3.3 Performance when applied separately to plant/animal associated bacterial effectors

The different results on the test and the *Xanthomonas* datasets led us to hypothesize that plants and animal bacterial pathogens may have different type III secretion signals. To test this hypothesis, we further divided our training and testing data to samples derived from plant pathogens and from animal pathogens (see Methods). We then evaluated the models trained on the different training sets, on the following test sets: T3Es derived from animal pathogens, T3Es derived from plant pathogens, T3Es derived from both plant and animal pathogens, and T3Es derived from *Xanthomonas*. Our results show that predicting T3Es from animal pathogens is best achieved by training the model on animal-derived T3Es, and similarly for plant pathogens (**Table 4**). Furthermore, as expected, the best predictor for *Xanthomonas* T3Es is the model trained on the T3Es from the plant pathogens. These results suggest that the secretion signal of animals and plants are different to some extent.

**Table 4:** The performance of the best set of features when applied separately to animal and plant associated bacterial effectors.

A					
Training data	Plant test dataset (40)	Animal test dataset (20)	Animal + plant test dataset (20 + 40)	Animal + plant test dataset (20 + 20)	*Xanthomonas*
**Animal pathogens T3Es (229)**	0.63 (0.65)	**0.77** **(0.78)**	0.69 (0.7)	0.7 (0.71)	0.44 (0.46)
**Plant pathogens T3Es (229)**	**0.8** **(0.8)**	0.71 (0.7)	**0.76** **(0.78)**	**0.77** **(0.76)**	**0.64** **(0.67)**
**Both animal and plant pathogens T3Es (115 + 114)**	0.53 (0.54)	0.55 (0.56)	0.6 (0.63)	0.62 (0.61)	0.58 (0.58)
**B**					
Training data	Plant test dataset (40)	Animal test dataset (20)	Animal + plant test dataset (20+40)	Animal + plant test dataset (20+ 20)	*Xanthomonas*
**Animal pathogens T3Es (229)**	0.63 (0.64)	0.77 (0.76)	0.69 (0.71)	0.7 (0.71)	0.44 (0.46)
**Plant pathogens T3Es (268)**	0.8 (0.8)	0.71 (0.72)	0.76 (0.77)	0.77 (0.77)	0.64 (0.66)
**Both animal and plant pathogens T3Es (497)**	**0.86** **(0.88)**	**0.79** (0.72)	**0.83** **(0.85)**	**0.84** **(0.85)**	**0.72** **(0.73)**

(A): each training data include 229 T3Es; (B): the training data include the maximum possible T3Es from that category. In bold is the best training data for each testing data. Data sizes are in parenthesis. The scores are MCC and AUPRC (in parenthesis).

In the above results, we compared plant versus animal models that were trained on the same number of effectors. We next asked whether the plant-based model can benefit from the inclusion of animal T3Es and similarly, whether the animal-based model can benefit from the inclusion of plant T3Es. Our results clearly show that the accuracy is increased when the largest number of positive samples is considered (**Table 4**). These results suggest that despite evidential differences between secretion signals of plant and animal pathogens, a model extracting information from all known effectors better captures the secretion signals, compared to a more specialized model trained on a smaller number of effectors.

### 3.4 Integrating the secretion signal feature into the Effectidor web server and running example on *Ralstonia solanacearum* GMI1000

We next tested the effect of integrating the secretion signal score based on the LME model and the Hobbs et al. (2016) features on the performance of Effectidor. This effect was evaluated on the plant pathogen *Ralstonia solanacearum* GMI1000. To separate training from testing data and to mimic the applicability of Effectidor to detect T3Es in a newly sequenced bacterium, we removed all *Ralstonia* effectors from the database of known T3Es that is used within the Effectidor web server for homology searching (see Methods). We ran Effectidor twice, with or without the additional feature of the signal score. To obtain the signal score for this testing case, we trained the model based on the non-redundant T3Es data, as described in the methods, excluding *Ralstonia* data.

**Figure 3:**
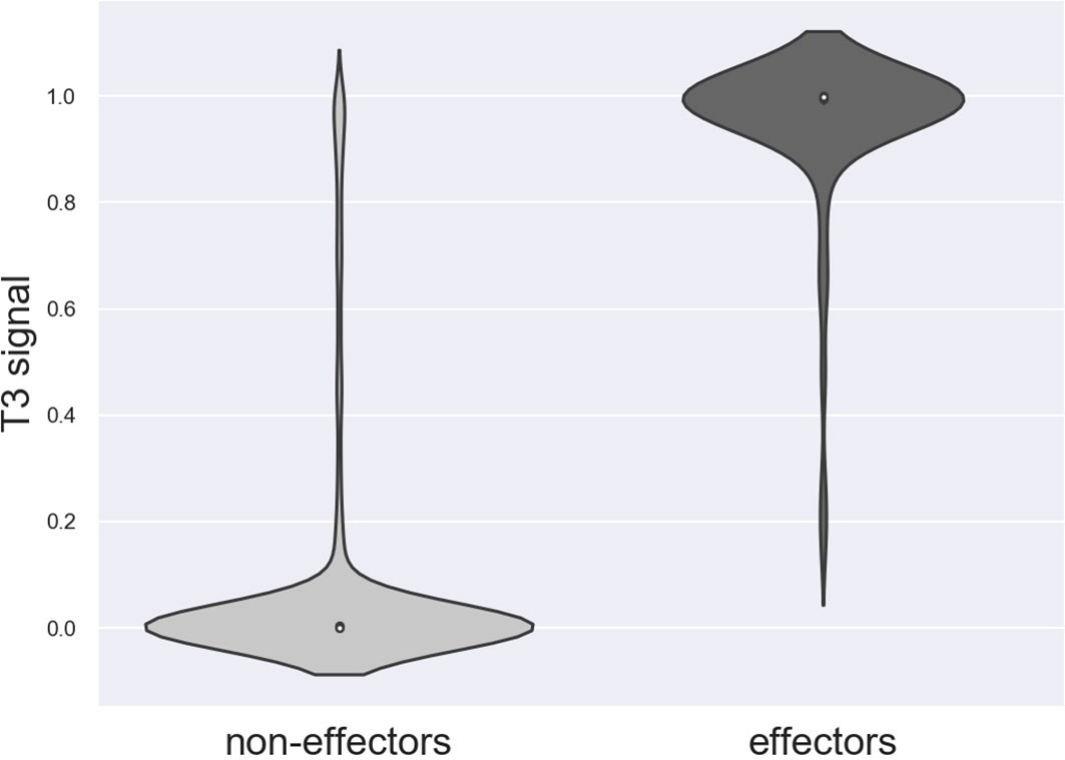
Distribution of the secretion signal score among the 2,661 non-effectors and among the 80 effectors in *R. solanacearum* GMI1000.

Nine effectors were found to harbor homology to T3Es outside *Ralstonia* and were considered as the positive trainingset for Effectidor. The inclusion of the secretion signal feature substantially increased performance: the confusion matrices are given in **Table 5**. The MCCs with and without this featurewere 0.72 and 0.65, respectively, while the AUPRC with and without this feature were 0.94 and 0.93, respectively. The newly added feature was found to be highly informative (**Figure 3**).

**Table 5:** The effect of including a novel feature that quantifies the strength of the secretion signal on the performance of Effectidor.

	A			B			C		
		T3Es	Non-T3Es		T3Es	Non-T3Es		T3Es	Non-T3Es
**Predicted T3Es**		34	0		42	0		77	168
**Predicted non-T3Es**		46	2,661		38	2,661		3	2,493

**(A)**: without the secretion signal feature; **(B)**: with the secretion signal feature; **(C)**: the signal feature alone.

**Figure 4:**
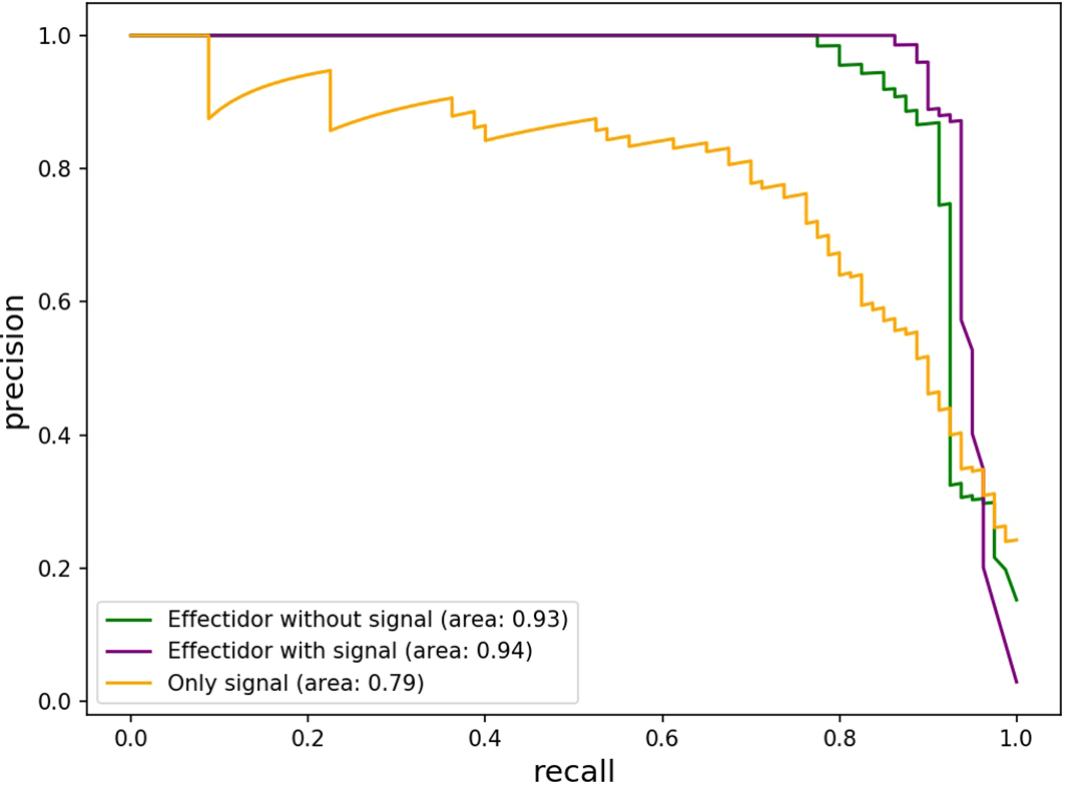
Precision-Recall Curves of predicting T3Es in *R. solanacearum* GMI1000 based on the secretion signal feature as a sole feature, and based on the predictions of Effectidor, with and without the inclusion of the secretion signal feature.

Using this feature alone, the achieved AUPRC and MCC were 0.79 and 0.53, respectively. Despite these low scores compared to the scores achieved by Effectidor, only three effectors out of 80 T3Es known in this strain were misclassified as non-effectors by this feature. Specifically, RS_RS23105 (RipAR, formerly Rip61), RS_RS10690 (RipS6, formerly SKWP6), and RS_RS26010 (RipBM) signal scores were 0.477, 0.252, and 0.166, respectively. The latter is reported to be present only as a pseudogene in *R. solanacearum* GMI100 in the “Ralstonia T3E” dataset (https://iant.toulouse.inra.fr/T3E) (Peeters et al., 2013). However, classification based only on the secretion signal score led to a high number of false-positives: 168 non-effectors were classified as effectors, i.e., they obtained a signal score higher than 0.5 (**Table 5**). The Precision-Recall curves, of Effectidor and of the signal feature alone, are available in **Figure 4**.

### 3.5 Interpreting the secretion signal using attention maps

**Figure 5:**
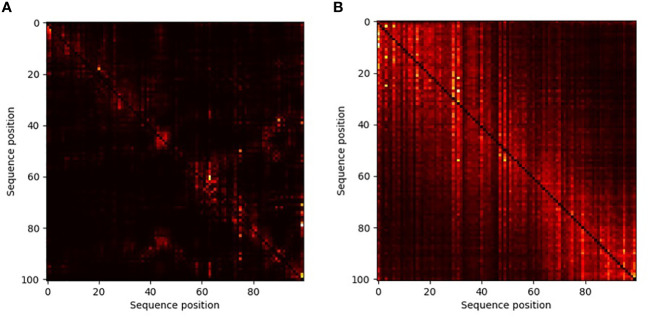
Attention maps of non-T3Es **(A)** and T3Es **(B)** demonstrating the effect of different positions in the amino acids sequence in the embedding process. The lighter the color, the more significant the interaction is. In these maps, each column (j) is a position in the sequence, and each entry (i,j) is the effect of position i on position j. The diagonal, which reflects the effect of each position on itself, was blacked out.

In transformer-based neural networks, a key component is the attention mechanism (Vaswani et al., 2017). This mechanism allows the model to learn which “words” attend to which other “words”. In our case the attention maps provide information regarding interactions among positions within the secretion signal. This, in turn, allows us to better understand, for each position within a specific sequence, which other positions are most important for the embedding. By contrasting the average attention matrix across all positive versus the average matrix over all negative sequences, we reviled different interactions among sites between T3E versus non-effectors (**Figure 5**). In the positive sequences, a large number of interactions among positions across the sequence is observed, even though there is strong variability of amino acids in each position. In contrast, when embedding the negative sequences, each position does not attend to many other positions. This analysis shows the diffusive nature of the type III secretion signal and warrants further research into each of these multi-position interactions.

## 4 Discussion

In this study, we aimed to better characterize the secretion signal of T3Es. We developed a novel NLP-based approach, using transformers that were specially derived for capturing information in protein MSAs, and demonstrated that classification based on this approach is more accurate than previous approaches. All models were compared using T3Es from various bacteria, including both plant and animal pathogens. Finally, we integrated this feature as part of the Effectidor web server for predicting T3Es.

The modeling of biological sequence data as a language and the incorporation of ML tools for languages, led to many advances in various biological domains (Rao et al., 2020; Rives et al., 2021; Trotter et al., 2021). Naturally, the problem of classifying proteins to either T3Es or non-effectors can also be approached using NLP-based methodologies. The first effort in this direction, from Fu and Yang (2019) was based on the now classic algorithm called Word2Vec, specifically, the Skip-Gram version with negative sampling (Mikolov et al., 2013). This algorithm embeds each possible protein sequence in a high-dimension space. The coordinates of this vector are used as features in standard classification algorithms. When adapting Word2Vec to protein sequences, each k-mer sequence is a word, and the entire sequence is represented as multiple overlapping k-mers. The embedding is done so that k-mers that appear together are close to each other in the abovementioned space. The final sequence embedding is obtained by averaging all k-mer embeddings. To learn this embedding protein sequences in this space, Fu and Yang (2019) analyzed a corpus of 25 million proteins from the UNIPROT50 data.

The Word2Vec algorithm clearly advanced the field of NLP in general. Since its publication, more advanced NLP algorithms were developed. These algorithms enable more efficient and accurate embedding of biological sequences. Among these advanced algorithms is Facebook’s MSA-transformer (Rao et al., 2021). Unlike Word2Vec, this algorithm applies large, transformer-based language models, which were trained on the entire RefSeq protein data. One advantage of this transformer-based algorithm is that unlike Word2Vec, there is no need to “parse” protein sequences into “words”, which do not appear naturally in sequence data, i.e., the transformer-based method of embedding allows to consider relations between amino acids across the entire protein sequence and not only a k-mer away. Of note, the LME approach developed here bypasses the need to explicitly design specific features.

When training and testing the algorithm, it is important to determine which proteins are included as negatives and positives. First, we note that in most applications of ML-based algorithms for effector identification, the input is the entire set of proteins encoded in a given bacterial genome. In such a scenario, we expect a few positive instances (effectors) in a sea of non-effectors (the rest of the proteins). This motivated us to establish a benchmark dataset that includes thousands of non-effectors. We note that in some previous works, the set of negatives did not well reflect this scenario. For example, in EP3 (Li et al., 2021), the negative set was composed of effectors of different secretion systems, other than T3SS. Clearly, this negative set is a biased sample of non-T3Es. Moreover, in Bastion3 (Wang et al., 2019) and in T3Sepp (Hui et al., 2020) the negative and positive sets were of the same size. The negative dataset used in pEffect (Goldberg et al., 2016) was enriched with eukaryotic proteins, such that only 37% of the negative dataset were bacterial. Of note, in this work, we tested the possibility of using another bacterial genome that does not encode a secretion system to define the negative set (*Lysobacter capsici* strain 55). This yielded performance almost identical to that obtained when negatives were defined based on the *E. coli* K12 genome (not shown). Having an accurate list of positives is also important. Each T3E assumed to be positive in this work was tested by ensuring that it is encoded within a genome that encodes the T3SS. Using this criterion, we discovered that erroneous T3Es were included in previous studies. We provide both the non-redundant data that were used in this work, and the entire data, which include effectors with high sequence similarity, from which the non-redundant data were derived. Finally, each effector is associated with the pathogen from which it is derived, allowing to test the accuracy on different taxonomic groups. Our benchmark data are available at https://github.com/naamawagner/T3ES_secretion_signal_analysis/tree/main/data.

The effort to classify effectors based on their secretion signal alone, demonstrates our limited understanding of the secretion signal, i.e., the classification accuracy is a measure of our computational ability to characterize which proteins are recognized and secreted by the T3SS. Our results clearly show that despite years of progress in this field, we still do not have sufficiently accurate models of the secretion signals, and on test data, we still experience dozens of false positives and negatives. Why is the classification mediocre? Several computational explanations are possible. First, the performance may be improved by applying more advanced algorithms on the same data. Second, the training data may be too small and do not capture the true variety of T3Es. Third, it could be that some errors in the benchmark data exist, which reduces accuracy. For example, *E. coli* K-12 does not have a T3SS and therefore we assume that its proteins are not secreted. However, it is possible that if such proteins were introduced to a bacterium with a secretion system – they would be secreted, i.e., they are not true negatives. In support of this hypothesis, (Wang et al. 2013b) tested the translocation of yeast proteins that had secretion signal features in their N-terminus in *Salmonella*. It was shown that these yeast proteins were translocated, despite the fact that yeast lacks a T3SS.

When discussing secretion of T3Es it is important to mention the involvement of chaperones. Chaperones were shown to affect the secretion of some T3Es while other T3Es were often found to be unaffected by chaperones (Ernst et al., 2018). In addition, chaperone binding sites were found to reside in the N-terminus of effectors, but were also found to bind to regions beyond the first 100 N-terminal residues (Parsot et al., 2003). Moreover, while the secretion apparatus and possibly the secretion signal is shared among T3Es from both animal and plant associated bacteria, the chaperones and their binding sites are highly variable. It is highly possible that the secretion signal provides the main driving force for secretion, and the chaperones fine-tune the secretion process, e.g., by scheduling the order of secretion or by preventing their aggregation or degradation when stored within the bacterial cell (Parsot et al., 2003).

The challenge of computationally characterizing the secretion signal could be of great importance to the in-silico synthesis of new effectors. Several efforts have been done to use Transcription Activator-Like Effectors Nucleases (TALEN) and Transcription Activator-Like Effectors (TALE) for manipulating gene expression and for gene editing purposes (Yang et al., 2013; Gao et al., 2014). Such technological advances may improve crop production or cure genetic diseases. As stated by Cheng et al. (2021), TALEs have the potential to perform better for such tasks than CRISPR/Cas9 because TALEs recognize more specific DNA segments than the CRISPR/Cas9 system and thus they are less prone to mistakes. Moreover, they are also encoded on a shorter DNA sequence, which may facilitate their usage in various systems. Better elucidating the secretion signal of T3Es could assist the engineering of such secreted proteins.

As stated above, the underlying assumption in this work is that a universal secretion signal exists that characterizes all T3Es. In this study we have shown that learning the secretion signal from T3Es encoded in animal-associated pathogens can be used to identify T3Es in plant-associated pathogens and vice versa, although a slight reduction in prediction ability was observed in such comparisons. Given the diversity of T3SS among pathogenic bacteria, it is highly possible that many T3Es are clade specific. To our knowledge, translocation tests of plant-associated T3Es in animal-associated bacteria, and vice versa, have not been conducted in large numbers. Such experiments have the potential to reveal how universal the secretion signal is.

In this work we explored the utility of different ways to extract meaningful representation of sequence data in vector space. We tested these methods for the task of identifying T3Es. These methods can also be applied to many additional bioinformatics tasks that rely on the analysis of protein sequences, e.g., predicting type IV effectors (Lewis et al., 2019), predicting fungal effectors (Sperschneider et al., 2018), protein contact prediction (Fukuda and Tomii, 2020), and predicting protein localization (Peabody et al., 2020).

## Data availability statement

The original contributions presented in the study are included in the article/supplementary material. Further inquiries can be directed to the corresponding author.

## Author contributions

NW, NP, and TP conceived the project. MA and ED suggested, applied and tested the NLP approaches. NE developed the HMM model. BZ and MMP implemented some features and analyzed effector datasets. NW implemented all the classic ML approaches and integrated the new feature in Effectidor. All authors analyzed the results and helped writing the manuscript. All authors approved the final manuscript.

## Funding

NW, MA, NE, and ED were supported in part by a fellowship from the Edmond J. Safra Center for Bioinformatics at Tel Aviv University.

## Acknowledgments

Israel Science Foundation (ISF) [2818/21 to T.P.]; Edmond J. Safra Center for Bioinformatics at Tel Aviv University Fellowship to NW, MA, NE, and ED, TP’s research is supported in part by the Edouard Seroussi Chair for Protein Nanobiotechnology, Tel Aviv University.

## Conflict of interest

The authors declare that the research was conducted in the absence of any commercial or financial relationships that could be construed as a potential conflict of interest.

## Publisher’s note

All claims expressed in this article are solely those of the authors and do not necessarily represent those of their affiliated organizations, or those of the publisher, the editors and the reviewers. Any product that may be evaluated in this article, or claim that may be made by its manufacturer, is not guaranteed or endorsed by the publisher.
